# Doppler Radar Vital Signs Detection Method Based on Higher Order Cyclostationary

**DOI:** 10.3390/s18010047

**Published:** 2017-12-26

**Authors:** Zhibin Yu, Duo Zhao, Zhiqiang Zhang

**Affiliations:** 1 School of Electrical Engineering, Southwest Jiaotong University, Chengdu 610031, China; zhaoduo@swjtu.cn; 2 School of Electrical and Electronic Engineering, University of Leeds, Leeds LS2 9AY, UK; z.zhang3@leeds.ac.uk

**Keywords:** vital signs, signal processing, heart and respiration rate, higher order cyclostationary, Doppler radar

## Abstract

Due to the non-contact nature, using Doppler radar sensors to detect vital signs such as heart and respiration rates of a human subject is getting more and more attention. However, the related detection-method research meets lots of challenges due to electromagnetic interferences, clutter and random motion interferences. In this paper, a novel third-order cyclic cummulant (TOCC) detection method, which is insensitive to Gaussian interference and non-cyclic signals, is proposed to investigate the heart and respiration rate based on continuous wave Doppler radars. The *k*-th order cyclostationary properties of the radar signal with hidden periodicities and random motions are analyzed. The third-order cyclostationary detection theory of the heart and respiration rate is studied. Experimental results show that the third-order cyclostationary approach has better estimation accuracy for detecting the vital signs from the received radar signal under low SNR, strong clutter noise and random motion interferences.

## 1. Introduction

Due to the non-contact nature, vital signs monitoring systems using radar sensors have been introduced in a wide range of applications, such as search and rescue operations, security and health care [1,2,3]. This technology is able to remotely monitor vital signs of a human subject by analyzing received radar signals. The concepts and advantages of non-contact vital signs detection have been demonstrated by pioneers [4,5]. The use of radar systems for the detection vital signs can be dated back to the 1970s [4]. Subsequent work in this area main focuses on improving the performance of detection method. In order to estimate the heart rate, a novel detection method, based on the continuous wavelet transform (CWT), was proposed to process the Doppler radar signal [6]. However, the method has limited resolution in both time and frequency and depends on fixed basis functions. While a time-window variation technique [7] guarantees a sufficient frequency spectrum resolution for the heart rate measurement, it is difficult to estimate the respiration rate simultaneously due to the respiration harmonic interference. The software for automated detection was demonstrated by adaptive cancellation of respiration harmonics [8]. But this method requires a priori knowledge of the basis frequency of the respiration signal. Empirical mode decomposition (EMD) decomposes adaptively a signal into a set of AM/FM components termed as intrinsic mode functions (IMFs) without the prior knowledge [9]. Accordingly, EMD-based Doppler radar data analysis has been successfully applied in heart and respiration rates detection of human subject [10,11]. However, the use of cubic splines and Hilbert transform in EMD process results in negative frequencies and a loss of amplitude and frequency information [12], and the decomposed results are affected by the harmonics of the respiration and heart. By using multiple higher order cumulants to reduce the affection of harmonics, the heart and respiration rates were estimated for the ultrawideband (UWB) radar signal [13]. However, the estimation accuracy is significantly degrade due to strong clutters and random body movements.

Recent years, the phase compensation method was also been explored to suppress random body movements [14]. In order to solve the null point and nonlinear issues in small-angle approximation-based Doppler radar sensors and eliminate the codomain restriction in the arctangent demodulation approach, an extended differentiate and crossmultiply (DACM) algorithm was proposed [15]. Based on stepped-frequency continuous wave radar, a spectrum analysis method [16] and a state space method [17] were applied to detect cardiac and respiratory rates, and much interest has been paid to using a single-continuous wave Doppler radar to detect heartbeat and respiration rates [18,19]. In order to enhance the accuracy of the heart rate estimation in strong clutter noise, a phase-based methods was proposed by using UWB impulse Doppler radar [20]. While the accuracy of the heart and respiration rates have been improved for relatively stationary and isolated subjects by applying the above methods, it is still a main challenge to obtain reliable data in the presence of human random body motion and other moving objects around. Based on the periodic chest displacement due to the periodic heartbeat and breathing, and the nonperiodic random subject motion, a second-order cyclostationary model [21,22] was successfully applied in the heart and respiration rates detection field. However, the second-order cyclostationary method is invalid for output signal that is usually proportional to the periodic chest displacement summed [23]. As with most of the proposed detection methods [14,15,17,18,19,20], this method is only available for complex signal demodulation (CSD). While CSD solves the null point problem, it generates harmonics [7].

Over the past few decades, lots of successful non-contact detection methods for the heart and respiration rates based on different conditions have been proposed. From the point of view of the radars, these methods can be roughly divided into three categories: UWB radar detection method [6,13,14,20], continuous-wave (CW) Doppler radar detection method [8,10,11,15,19,21,22], and frequency-modulated continuous-wave (FMCW) radar detection method [16,17]. Compare to UWB radar and FMCW radar, CW Doppler radar has drawn more research interests due to the advantages of low power consumption, large detection range and simple radio architecture [18]. Because of different hardware architectures, the detection performances of different radars are different. Meanwhile, the proposed signal processing method often focuses on a particular issue under a particular condition. Therefore, these proposed detection methods have their own advantages and shortcomings. Although non-contact vital sign measurement has been studied for many years and lots of successful methods have been reported, the impacts of random subject movement, harmonic interferences and clutter noise are still challenging issues in non-contact vital signs detection field.

In this paper, we propose a higher order cyclostationary (HOCS) approach to detect the heart and respiration rate based on CW Doppler radar. The received radar signals includes harmonic interferences, random subject movements and clutter noise. The heart and respiration signals can be modeled as a cyclostationary process or almost cyclostationary process [21,22]. The received radar signal usually has a cyclic pattern with statistical properties. Cyclostationary theory can also be used to analyze the signals with hidden periodicities by its cyclic statistics properties. Therefore, the cyclostationary theory is one of the most suitable methods. According to the cyclostationary theory, HOCS of any Gaussian signal are zero theoretically, as a result in the new method is insensitive to Gaussian interferes (Gaussian noise and Gaussian clutter) [24,25]. For non-cyclic signal, HOCS has still the insensitive property when the cyclic frequency value is non-zero.

We mainly focus on the theoretical and experimental vital sign detection analysis based on the third-order cyclostationary method. We proved the third-order cyclic cummulant (TOCC) is applicable to detect the heart and respiration rates in the Doppler radar vital sign monitoring system. In order to verify the detecting performances of the proposed method, a series experiments in different situations (Gaussian noise, subject movement interferences, holding breath, changing the distance between the surface of antenna and the human chest) are carried out.

## 2. Materials and Methods

### 2.1. Doppler Radar Vital Sign Model

In signal processing of Doppler radar vital signs monitoring system, the received signal contains a frequency shift proportional to the target speed (Doppler Effect). Suppose the detected target is human thorax, Doppler echo signal includes possible the respiration and heartbeat information due to the chest motion caused by respiration and heartbeat. The respiration frequency is usually in the range 0.1–0.8 Hz and the heartbeat frequency in the range 0.8–2 Hz [3].

Given CW Doppler radar system that transmits a single tone signal at frequency *f*. Neglecting amplitude variations, the transmitted signal can be expressed as [3]

(1) s(t)=cos(2πft+ϕ(t))

where ϕ(t) is phase noise of the oscillator. This signal is reflected from the human subject at a distance *d*, with a time-varying displacement given by *x*(*t*). The reflected signal is amplitude and frequency modulated by motion of object, and x(t)<<d. Neglecting amplitude variations, initial phase offset and noise, the received signal *R*(*t*) can be obtained as [23]

(2) $$R\left(t\right)=\cos[2\pi ft-\frac{4\pi d}{\lambda }-\frac{4\pi x(t)}{\lambda }+\phi \left(t-\frac{2d}{c}\right)]$$

where λ is wavelength, *c* denotes the velocity of light. According to Doppler Effect, the received signal is modulated by the periodic motion of the target. The periodic target motion information can be demodulated if this signal is multiplied by a local oscillator signal that is derived from the same source as the transmitted signal [23]. Therefore, when Doppler radar use the same oscillator for the transmitter and local oscillator, and the received *R*(*t*) and local oscillator signals are mixed, the baseband output signal of the monitoring system can be expressed as [23]

(3) $$B\left(t\right)=\cos(\theta +\frac{4\pi x(t)}{\lambda }+\Delta \phi \left(t\right))$$

where

$$\theta =\frac{4\pi d}{\lambda }+\theta_{0}$$

is the constant phase shift and Δϕ(t) is the residual phase noise, and it can be computed as

$$\Delta \phi \left(t\right)=\phi \left(t\right)-\phi \left(t-\frac{2d}{c}\right).$$

Based on the Doppler Effect, the radio frequency wave reflected from the surface of the subjects chest undergoes two main phase shifts proportional to the surface displacement due to heartbeat and respiration affect chest periodic motions. The quasi-periodic motions can be modeled as [13,18,22]

(4) $$x\left(t\right)=a_{r}\cos\left(2\pi f_{r}t\right)+a_{h}\cos\left(2\pi f_{h}t\right)$$

where $a_{r}$ and $a_{h}$ are related to the vibration amplitude of respiration and heartbeat, respectively, and $f_{r}$ and $f_{h}$ correspond to frequencies of respiration and heartbeat, respectively.

The motion-modulated signal reflected from the random subject movement can be modeled as a one-dimensional random process with uniform distribution in the specified interval [21]. Consider the receiver noise, by introducing (4) into (3), the baseband output can be expressed as

(5) $$\begin{array}{cc} B(t) & =A\cos\left\{\theta +A_{r}\cos\left(2\pi f_{r}t\right)+A_{h}\cos\left(2\pi f_{h}t\right)+\frac{4\pi x_{I}\left(t\right)}{\lambda }+\Delta \phi \left(t\right)\right\}+N\left(t\right) \end{array}$$

where

$$A_{r}=\frac{4\pi }{\lambda }a_{r},A_{h}=\frac{4\pi }{\lambda }a_{h},$$

$x_{I}\left(t\right)$ and N(t) denote the random subject movement and the receiver noise, respectively, and these signals are assumed real and mutually independent. In complex number space, we rewritten (5) as

(6) $$\begin{array}{cc} B(t) & =Aexp\left\{j[\theta +A_{r}\cos\left(2\pi f_{r}t\right)+A_{h}\cos\left(2\pi f_{h}t\right)+\frac{4\pi x_{I}\left(t\right)}{\lambda }+\Delta \phi \left(t\right)]\right\}+N_{Z}\left(t\right). \end{array}$$

Equation (6) can be re-expressed in (7) based on Bessel series properties:

(7) $$\begin{array}{cc} B(t) & =A\sum_{n=-\infty }^{\infty }\sum_{m=-\infty }^{\infty }J_{n}\left(A_{r}\right)J_{m}\left(A_{h}\right)exp\left\{j[2n\pi f_{r}t+2m\pi f_{h}t+\frac{4\pi x_{I}\left(t\right)}{\lambda }+C+\Delta \phi \left(t\right)]\right\}+N_{Z}\left(t\right) \end{array}$$

where *C* and $N_{Z}\left(t\right)$ indicate the *dc* value and complex noise, respectively.

We re-rewrite (7) as

(8) $$\begin{array}{cc} B(t) & =DI\left(t\right)\sum_{n=-\infty }^{\infty }\sum_{m=-\infty }^{\infty }J_{n}\left(A_{r}\right)J_{m}\left(A_{h}\right)exp\left[j\left(2n\pi f_{r}t+2m\pi f_{h}t\right)\right]+N_{Z}\left(t\right) \end{array}$$

where

D=Aexp(jC)

and

$$I\left(t\right)=exp\left[j\frac{4\pi }{\lambda }x_{I}\left(t\right)\right]exp\left[j\Delta \phi \left(t\right)\right].$$

By referring to literature [21], the Fourier spectrum of (8) can be obtained as

(9) $$\begin{array}{cc} S_{B}\left(f\right) & =DI\left(f\right)\sum_{n=-\infty }^{\infty }\sum_{m=-\infty }^{\infty }J_{n}\left(A_{r}\right)J_{m}\left(A_{h}\right)delta\left[f-\left(nf_{r}+mf_{h}\right)\right]+N_{Z}\left(f\right). \end{array}$$

From (9), the discrete spectrum of baseband output signal consists of respiration frequency $f_{r}$, heartbeat frequency $f_{h}$ and all sums and differences of their harmonics.

### 2.2. Cyclostationary Detection Theory

One of the goals, for detecting vital signs by using Doppler radar, is to estimate heart and respiration rates from the received radar signal. According to the above analysis, the received radar signal exhibits cyclostationarity. Therefore, in this Section, we propose a novel vital sign detection method based on third-order cyclostationary analysis for the Doppler radar vital sign signal model.

The time varying *k*-th order moment of a cyclostationary sign *s*(*t*) can be defined as [25,26]

$$m_{ks}\left(t;\tau \right)=E\left\{s\left(t\right)s^{*}\left(t+\tau_{1}\right)\cdots s^{*}\left(t+\tau_{k-1}\right)\right\}$$

where ∗ denotes an optional conjugation. The time-varying *k*-th order moments can be further expressed in terms of its lag-dependent Fourier coefficients as

$$m_{ks}\left(t;\tau \right)=\sum_{\alpha }M_{ks}\left(\alpha ;\tau \right)e^{-j\alpha t}$$

where α is a cycle frequency and the Fourier coefficients $M_{ks}\left(\alpha ;\tau \right)$ is the *k*-th order cyclic moments and it satisfy [26]

(10) $$M_{ks}\left(\alpha ;\tau \right)=\lim_{T\to \infty }\sum_{t=0}^{T-1}m_{ks}\left(t;\tau \right)e^{-j\alpha t}$$

where *T* is the average length of time. Similarly, the *k*-th order cyclic cumulant $C_{ks}\left(\beta ;\tau \right)$ is also the Fourier coefficients of the time-varying *k*-th order cumulant $c_{ks}\left(t;\tau \right)$ can be represented as

(11) $$C_{ks}\left(\beta ;\tau \right)=\lim_{T\to \infty }\frac{1}{T}\sum_{t=0}^{T-1}c_{ks}\left(t;\tau \right)e^{-j\beta t}$$

where

$$c_{ks}\left(t;\tau \right)=\sum_{\beta }C_{ks}\left(\beta ;\tau \right)e^{-j\beta t}.$$

The conversion relations between the cyclic moments and the cyclic cumulants found in the higher order statistics theory can be expressed as follow in [24,26]

(12) $$C_{s}{\left(\beta ;\tau \right)}_{k}=\sum_{D_{k}}\left[{\left(-1\right)}^{p-1}\left(p-1\right)!\sum_{\alpha^{T}1=\beta }\prod_{j=1}^{p}M_{s}{\left(\alpha_{j};\tau_{u_{j}}\right)}_{k_{j}}\right]$$

where $D_{k}$ denotes the distinct partitions of the index set {1,2,…,k}, *p* is the number of elements in a partition, and the set of indices belonging to a partition is indicated by $u_{j}\;j\in 1,2,\ldots ,p$, and $\alpha ={\left[\alpha_{1}\ldots \alpha_{p}\right]}^{T}$ whose entries sum to β and satisfy $M_{s}{\left(\alpha_{j};\tau_{u_{j}}\right)}_{k_{j}}\neq 0$ for j=1,2,…,p. Then given the cyclic moments of a signal, one can use (12) to compute its corresponding cyclic cumulants.

From (10)–(12), for a zero mean value signal s(t), the second cyclic cumulant and the third-order cyclic cumulant (TOCC) are both equivalent to their respective moments. Therefore, we have

(13a) $$\begin{array}{cc} C_{2s}\left(\alpha ;\tau \right) & =M_{2s}\left(\alpha ;\tau \right) \end{array}$$

(13b) $$\begin{array}{cc} C_{3s}\left(\alpha ;\tau \right) & =M_{3s}\left(\alpha ;\tau \right). \end{array}$$

The Fourier transform of (13) can be expressed as

(14a) $$\begin{array}{cc} S_{2s}\left(\alpha ;f\right) & =\sum_{\tau }C_{2s}\left(\alpha ;\tau \right)e^{-j2\pi f\tau } \end{array}$$

(14b) $$\begin{array}{cc} S_{3s}\left(\alpha ;f\right) & =\sum_{\tau }C_{3s}\left(\alpha ;\tau \right)e^{-j2\pi f\tau }. \end{array}$$

**Figure 1:**
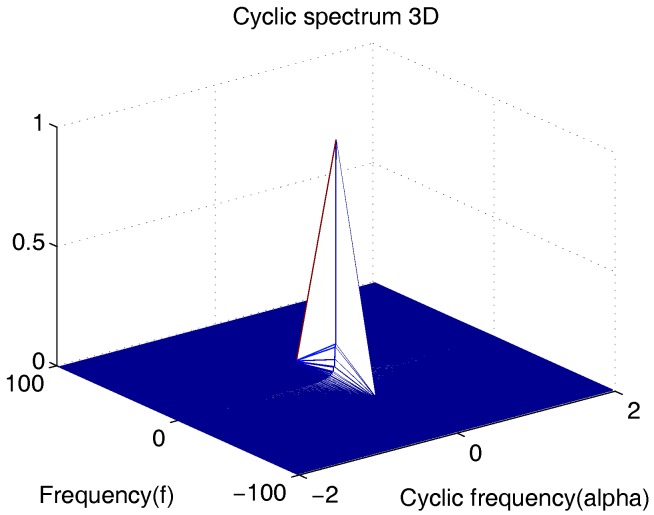
Spectral correlation function (SCF) of the second-order cyclostationary signal B(t) in (8), where noise and subject movement interferences are ignored.

According to (8), the discrete Spectral Correlation Function (SCF) of the second-order cyclostationary of the received radar signal can be expressed as follow in [21].

(15) $$\begin{array}{cc} S_{2B}\left(\alpha ;f\right) & =A^{2}{ℜ}_{I}\left(f\right)\sum_{n=-\infty }^{\infty }\sum_{m=-\infty }^{\infty }\sum_{k=-\infty }^{\infty }\sum_{l=-\infty }^{\infty }\sum_{\tau }J_{n}\left(A_{r}\right)J_{m}\left(A_{h}\right)J_{k}\left(A_{r}\right)J_{l}\left(A_{h}\right)e^{-j2\pi (kf_{r}+lf_{h}+f)\tau }\\ & \;\times \delta \left\{\alpha -\left[\left(n-k\right)f_{r}+\left(m-l\right)f_{h}\right]\right\}+C_{2N_{Z}\left(f\right)} \end{array}$$

where ${ℜ}_{I}\left(f\right)$ function indicates the Fourier transform of the subject random motion and phase noise, the terms of $C_{2N_{Z}\left(f\right)}$ which is SCF of $N_{Z}\left(\tau \right)$. Accordingly, the heart and respiration rate were estimated by the received Doppler radar signal in [21]. However, when $\tau =\tau_{0}$, n=k and m=l in (15), $S_{2B}\left(\alpha ;f\right)=0$ at α≠0. This suggested that heart and respiration rate may not be obtained by using the second order cyclostationary approach when the received radar signal includes only the first harmonic. In Figure 1, we depict one realization of the second-order cyclostationary signal B(t) in (8) for a carrier frequency of 2.4 GHz, where the $a_{h},a_{r},f_{h}\;and\;f_{r}$ values in this example are assumed as 0.06 cm, 0.15 cm, 1.4 Hz and 0.3 Hz, respectively. As shown in Figure 1, there is no visible peak along the cyclic frequency axis when α≠0.

Using (8), (11) and (13), the third-order cyclic cumulant of B(t) can be computed as

(16) $$\begin{array}{cc} C_{3s}\left(\alpha ;\tau \right)\; & =M_{3s}\left(\alpha ;\tau \right)\\ \; & =\lim_{T\to \infty }\frac{1}{T}\sum_{t=0}^{T-1}B\left(t\right)B\left(t+\tau_{1}\right)B^{*}\left(t+\tau_{2}\right)e^{-j2\pi \alpha t}\\ & =\lim_{T\to \infty }\frac{1}{T}\sum_{t=0}^{T-1}D^{3}C_{3I}\left(\tau \right)\\ & \;\times \sum_{n=-\infty }^{\infty }\sum_{m=-\infty }^{\infty }\sum_{k=-\infty }^{\infty }\sum_{l=-\infty }^{\infty }\sum_{o=-\infty }^{\infty }\sum_{p=-\infty }^{\infty }\;J_{n}\left(A_{r}\right)J_{m}\left(A_{h}\right)J_{k}\left(A_{r}\right)J_{l}\left(A_{h}\right)J_{o}\left(A_{r}\right)J_{p}\left(A_{h}\right)\\ & \;\times e^{j2\pi \left(kf_{r}+lf_{h}\right)\tau_{1}-j2\pi \left(of_{r}+pf_{h}\right)\tau_{2}}\times e^{-j2\pi \{\alpha -\left[\left(n+k-o\right)f_{r}+\left(m+l-p\right)f_{h}\right]\}t}+C_{3N_{Z}}\left(\alpha ;\tau \right) \end{array}$$

where $C_{3I}\left(\tau \right)$ function, which is the third-order cumulant of the motion and phase noise, acts as multiplicative noise for the delta functions, and the term of $C_{3N_{Z}}\left(\alpha ;\tau \right)$ is the third-order cyclic cumulant of Gaussian noise, and its value is zeros due to the higher order cyclic cumulant property.

Let $\tau_{2}=\tau_{1},\tau_{1}=\tau$ , the spectral function of the slice of the third-order cyclostationary signal B(t) is the Fourier transform of (16), and is obtained as

(17) $$\begin{array}{cc} S_{3B}\left(\alpha ;f\right)\; & =D^{3}C_{3I}\left(f\right)\sum_{n=-\infty }^{\infty }\sum_{m=-\infty }^{\infty }\sum_{k=-\infty }^{\infty }\sum_{l=-\infty }^{\infty }\sum_{o=-\infty }^{\infty }\sum_{p=-\infty }^{\infty }\sum_{\tau }\;J_{n}\left(A_{r}\right)J_{m}\left(A_{h}\right)J_{k}\left(A_{r}\right)J_{l}\left(A_{h}\right)J_{o}\left(A_{r}\right)J_{p}\left(A_{h}\right)\\ & \;\times e^{j2\pi [\left(k-o\right)f_{r}+\left(l-p\right)f_{h}-f]\tau }\times \delta \left\{\alpha -\left[\left(n+k-o\right)f_{r}+\left(m+l-p\right)f_{h}\right]\right\} \end{array}$$

where $S_{3B}\left(\alpha ;f\right)$ is a 2-D function that involves all of the frequencies along the cyclic axis. From (17), $S_{3B}\left(\alpha ;f\right)\neq 0$ when $\tau =\tau_{0}$ and α≠0. Therefore, the signal in (8) is a third-order cyclostationary signal.

For Doppler radar signals, the baseband output (3) can be approximately expressed by (18) when θ in (3) is an odd multiple of $\frac{\pi }{2}$ and x(t)≪λ [23].

(18) $$B\left(t\right)\approx \frac{4\pi x(t)}{\lambda }+\Delta \phi \left(t\right).$$

Considering the subject random movement I(t) and Gaussian noise N(t), we re-express (18) as

(19) B(t)=S(t)+I(t)+N(t)

where

$$I\left(t\right)=\frac{4\pi x_{I}\left(t\right)}{\lambda }+\Delta \phi \left(t\right),$$

S(t), and N(t) are the pair wise independence signals. From the aforementioned analysis, the vital sign signal is composed primarily of respiration, heartbeat and all sums and differences of their harmonics. Accordingly, S(t) can be expressed as

(20) $$S\left(t\right)=\sum_{n=-\infty }^{\infty }\sum_{m=-\infty }^{\infty }A_{nm}\cos\left[2\pi \left(nf_{r}+mf_{h}\right)t\right]$$

where $A_{nm}$ is the amplitude corresponding to the signal with the frequency $\left(nf_{r}+mf_{h}\right)$. For simplicity, we only analyse the first harmonic (i.e. n=1 and m=0; n=0 and m=1). Accordingly, S(t) can be rewritten as

(21) $$\begin{array}{cc} S(t) & =s_{r}\left(t\right)+s_{h}\left(t\right)\\ & =A_{r}\cos\left(2\pi f_{r}t\right)+A_{h}\cos\left(2\pi f_{h}t\right). \end{array}$$

According to the property of the *k*-th order cyclic cumulant [24,25], the *k*-th order cyclic cumulant of (19) can be given by

(22) $$C_{kB}\left(\alpha ;\tau \right)=C_{kS}\left(\alpha ;\tau \right)+C_{kI}\left(\alpha ;\tau \right)+C_{kN}\left(\alpha ;\tau \right).$$

The $C_{kI}\left(\alpha ;\tau \right)$ function, which is the *k*-th order cyclic cumulant of the motion and phase noise, acts as the dc component like the delta functions. The term $C_{kN}\left(\alpha ;\tau \right)$ is the *k*-th order cyclic cumulant of Gaussian noise. $C_{kN}\left(\alpha ;\tau \right)$ is autocorrelation functions of noise when k=2. If k>2, the value of $C_{kN}\left(\alpha ;\tau \right)$ is zero due to the property of higher order cyclic cumulant.

In (16) and (17), the third-order cyclostationary of the received signal retains the fundamental frequency along the cyclic axis. We calculate the third-order cyclic cumulant (TOCC) of (19) by using (23).

(23) $$C_{3B}\left(\alpha ;\tau \right)=C_{3S}\left(\alpha ;\tau \right)+C_{3I}\left(\alpha ;\tau \right)+C_{3N}\left(\alpha ;\tau \right).$$

The cyclic spectral function of the slice $\left(\tau_{2}=\tau_{1},\tau_{1}=\tau \right)$ of the third-order cumulant of B(t) can be written as

(24a) $$\begin{array}{c} \begin{array}{c} C_{3S}\left(\alpha ;\tau \right)\\ =\lim_{T\to \infty }\frac{1}{T}\sum_{t=0}^{T-1}S\left(t\right)S\left(t+\tau \right)S^{*}\left(t+\tau \right)e^{-j2\pi \alpha t}+C_{3I}\left(\alpha ;\tau \right)+0\\ =\lim_{T\to \infty }\frac{1}{T}\sum_{t=0}^{T-1}\left\{\left[A_{r}\cos\left(2\pi f_{r}t\right)+A_{h}\cos\left(2\pi f_{h}t\right)\right]\times {\left[A_{r}\cos\left(2\pi f_{r}\left(t+\tau \right)\right)+A_{h}\cos\left(2\pi f_{h}\left(t+\tau \right)\right)\right]}^{2}\right\}\\ \;\times e^{-j2\pi \alpha t}+C_{3I}\left(\alpha ;\tau \right). \end{array} \end{array}$$

In fact, time-delay τ is only closely related to the magnitude of estimated periodicity frequencies. Therefore, based on Euler’s formula, when τ=0, (24a) can be calculated as

(24b) $$\begin{array}{c} \begin{array}{c} C_{3S}\left(\alpha ;\tau \right)\\ =\langle K_{1}\left[e^{-j2\pi (\alpha -f_{r})t}+e^{-j2\pi (\alpha +f_{r})t}\right]+K_{2}\left[e^{-j2\pi (\alpha -f_{h})t}+e^{-j2\pi (\alpha +f_{h})t}\right]\\ \;+\frac{A_{r}A_{h}^{2}}{8}\left[3e^{-j2\pi (\alpha -\left(f_{r}+2f_{h}\right))t}+3e^{-j2\pi (\alpha +\left(f_{r}+2f_{h}\right))t}+3e^{-j2\pi (\alpha -\left(f_{r}-2f_{h}\right))t}+3e^{-j2\pi (\alpha +\left(f_{r}-2f_{h}\right))t}\right]\\ \;+\frac{A_{r}^{2}A_{h}}{8}\left[3e^{-j2\pi (\alpha -\left(2f_{r}+f_{h}\right))t}+3e^{-j2\pi (\alpha +\left(2f_{r}+f_{h}\right))t}+3e^{-j2\pi (\alpha -\left(2f_{r}-f_{h}\right))t}+3e^{-j2\pi (\alpha +\left(2f_{r}-f_{h}\right))t}\right]\\ \;+\frac{A_{r}^{3}}{8}\left[e^{-j2\pi (\alpha -3f_{r})t}+e^{-j2\pi (\alpha +3f_{r})t}\right]+\frac{A_{h}^{3}}{8}\left[e^{-j2\pi (\alpha -3f_{h})t}+e^{-j2\pi (\alpha +3f_{h})t}\right]\rangle_{t}+C_{3I}\left(\alpha ;0\right) \end{array} \end{array}$$

where $K_{1}=\frac{A_{r}A_{h}^{2}}{2}+\frac{A_{r}^{2}A_{h}+A_{h}^{3}}{4}+\frac{A_{r}^{3}}{8}$ and $K_{2}=\frac{A_{h}A_{r}^{2}}{2}+\frac{A_{h}^{2}A_{r}+A_{r}^{3}}{4}+\frac{A_{h}^{3}}{8}$. The spectral function of the slice of the third-order cyclostationary of B(t) is expressed as

(25) $$C_{3B}\left(\alpha ;f\right)=\sum_{\tau }C_{3S}\left(\alpha ;\tau \right)e^{-j2\pi f\tau }+C_{3I}\left(\alpha ;f\right).$$

Apparently, the term $C_{3B}\left(\alpha ;\tau \right)\neq 0$ when α≠0 in (24), i.e., B(t) has the third-order cyclostationarty property. As is obvious from (24b), there are corresponding spectral lines to cyclic frequencies $f_{r},-f_{r},3f_{r},-3f_{r},f_{h},-f_{h},3f_{h},-3f_{h},f_{r}+2f_{h},-\left(f_{r}+2f_{h}\right),f_{r}-2f_{h},-\left(f_{r}-2f_{h}\right),2f_{r}-f_{h},-\left(2f_{r}-f_{h}\right),2f_{r}+f_{h}$, and $-(2f_{r}+f_{h})$. These spectral lines are symmetric about the zero point. Therefore, for the radar signal in (5), we can detect heart and respiration frequencies with the third-order cyclic cyclostationary method. However new frequencies, such as $3f_{r},-3f_{r},3f_{h},-3f_{h},f_{r}+2f_{h},-\left(f_{r}+2f_{h}\right),f_{r}-2f_{h},-\left(f_{r}-2f_{h}\right),2f_{r}-f_{h},-\left(2f_{r}-f_{h}\right),2f_{r}+f_{h}$ and $-(2f_{r}+f_{h})$, are introduced in (24b). Although the energy of the single harmonic is little and the most of harmonic frequencies (excepting for $3f_{r},f_{h}-2f_{r}$ and $2f_{h}-f_{r}$) are over the ranges of the respiration and heartbeat frequencies, the detection of heartbeat and respiration rate may be interfered by the higher energy $3f_{r}$ signal when the introduced $3f_{r}$ signal has the same phase with the original third harmonic in the received signal.

**Figure 2:**
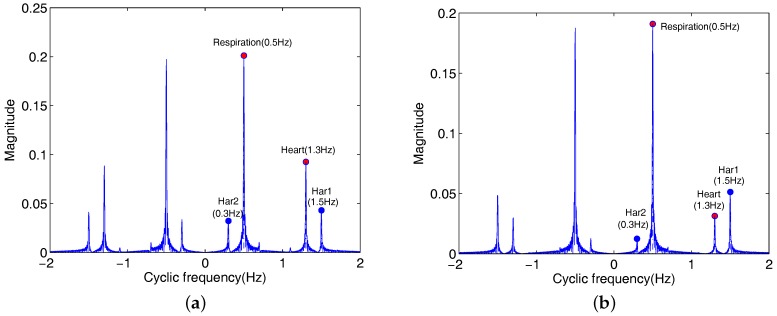
TOCC of B(t) in (5). (**a**) TOCC of the received signal; (**b**) TOCC of the received signal (here $a_{h}=0.02$ cm).

In Figure 2, it depicts one realization of the third-order cyclostationary of B(t) in (5), Here, carrier frequency of Doppler radar is 10.587 GHz (λ≈2.8 cm), and the $a_{h},a_{r},f_{h}$ and $f_{r}$ values in this example are assumed as 0.06 cm, 0.2 cm, 1.3 Hz and 0.5 Hz, respectively.

As is obvious from Figure 2a, the respiration and heartbeat frequencies estimated are equal to 0.4995 Hz and 1.301 Hz, respectively. Although the third harmonic (1.5 Hz) of the respiration and $f_{h}-2f_{r}=0.3$ Hz are cumulated, the magnitudes of harmonics are far below than the magnitudes of estimated frequencies. In this example, we replace only the value of $a_{h}$ with 0.02 cm. TOCC of B(t) in (5) is shown in Figure 2b. As is obvious from Figure 2b, the cumulated amplitude of $3f_{r}$ (1.5 Hz) is higher than the cumulated amplitude of $f_{h}$ (1.3 Hz). The mainly reason is that the introduced $3f_{r}$ signal is superimposed on the original third harmonic due to the same phase for two signals. After performing the third-order cyclostationary algorithm, the amplitude accumulated of the third harmonics is just more than the amplitude accumulated of heartbeat signal.

In order to reduce the third harmonic introduced in (24b), the received radar signal B(t) in (5) is projected into the Hilbert space, and the analytic signal of B(t) is formulated as

(26) $$y\left(t\right)=B\left(t\right)+jB_{H}\left(t\right)$$

where $B_{H}\left(t\right)$ is Hilbert transform of B(t). According to the property of Hilbert transform, $y_{I}\left(t\right)$ and $y_{N}\left(t\right)$ are complexity Gaussian signals. Values of $C_{3yI}\left(t\right)$ and $C_{3yN}\left(t\right)$ are zero in the theory. The analytic signal of s(t) is given as

(27) $$\begin{array}{cc} y_{S}\left(t\right) & =A_{r}\left[\cos\left(2\pi f_{r}t\right)+j\sin\left(2\pi f_{r}t\right)\right]+A_{h}\left[\cos\left(2\pi f_{h}t\right)+j\sin\left(2\pi f_{h}t\right)\right]\\ & =A_{r}e^{j2\pi f_{r}t}+A_{h}e^{j2\pi f_{h}t} \end{array}$$

where $\sin(2\pi f_{r}t)$ and $\sin(2\pi f_{h}t)$ is the Hilbert transform function of $\cos(2\pi f_{r}t)$ and $\cos(2\pi f_{h}t)$. The third-order cyclic cumulant of (26) can be calculated as

(28) $$\begin{array}{cc} C_{3y}\left(\alpha ;\tau \right)\; & =C_{3y_{S}}\left(\alpha ;\tau \right)+C_{3y_{I}}\left(\alpha ;\tau \right)+C_{3y_{N}}\left(\alpha ;\tau \right)\\ & \approx C_{3y_{S}}\left(\alpha ;\tau \right)\\ & =\lim_{T\to \infty }\frac{1}{T}\sum_{t=0}^{T-1}y_{S}\left(t\right)y_{S}\left(t+\tau \right)+y_{S}^{*}\left(t+\tau \right)e^{-j2\pi \alpha t}\\ & =\langle \left[A_{r}^{3}+A_{r}A_{h}^{2}+A_{r}A_{h}^{2}e^{j2\pi (f_{r}-f_{h})\tau }\right]e^{-j2\pi (\alpha -f_{r})t}\\ & \;+\left[A_{h}^{3}+A_{h}A_{r}^{2}+A_{h}A_{r}^{2}e^{j2\pi (f_{h}-f_{r})\tau }\right]e^{-j2\pi (\alpha -f_{h})t}\\ & \;+\left[A_{h}A_{r}^{2}e^{j2\pi (f_{r}-f_{h})\tau }\right]e^{-j2\pi [\alpha -\left(2f_{r}-f_{h}\right)]t}\\ & \;+\left[A_{r}A_{h}^{2}e^{j2\pi (f_{h}-f_{r})\tau }\right]e^{-j2\pi [\alpha -\left(2f_{h}-f_{r}\right)]t}\rangle_{t}. \end{array}$$

Performing the Fourier transform for (28), we have

(29) $$C_{3y}\left(\alpha ;f\right)=\sum_{\tau }C_{3y_{S}}\left(\alpha ;\tau \right)e^{-j2\pi f\tau }.$$

Clearly, from (28), the heart and respiration frequencies can be estimated when $a=f_{r}$ and $a=f_{h}$, and there is no new $3f_{r}$ introduced. Although cross terms $2f_{r}-f_{h}$ and $2f_{h}-f_{r}$ are introduced, the amplitude of the cross term is far below than $f_{r}$ or $f_{h}$ in (28).

**Figure 3:**
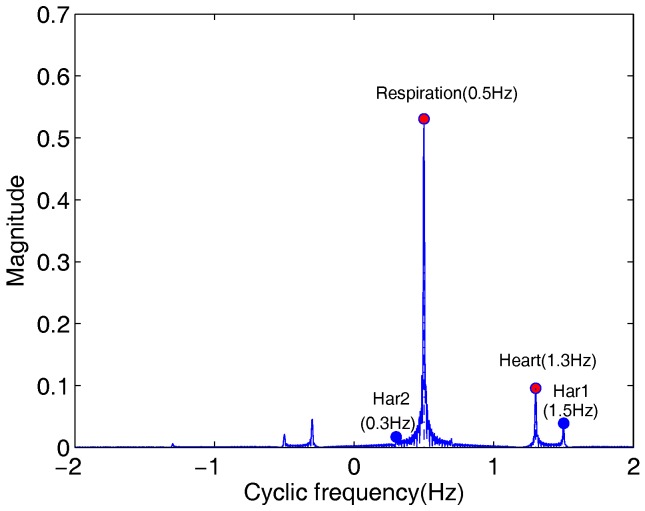
TOCC of Hilbert transform of B(t) with the same parameters with the ones in Figure 2b.

In Figure 3, it shows the TOCC of Hilbert transform of B(t) with the same parameters with Figure 2b. In Figure 3, the amplitude of the signal with the frequency 1.3 Hz is higher than the $3f_{r}$ (1.5 Hz). Comparing with Figure 2b, the new introduced frequency $3f_{r}$ in third-order cyclic cumulant is suppressed by using Hilbert transform method for the received signal B(t). The frequency 1.5 Hz in Figure 3 is the frequency of the original third harmonic $3f_{r}$ in the received radar signal B(t).

If the second-order cyclostationary method is used to analyse y(t) in (26) in Hilbert space, we have

(30) $$\begin{array}{cc} C_{2y}\left(\alpha ;\tau \right) & =C_{2y_{S}}\left(\alpha ;\tau \right)+C_{2y_{I}}\left(\alpha ;\tau \right)+C_{2y_{N}}\left(\alpha ;\tau \right)\\ & =C_{2y_{S}}\left(\alpha ;\tau \right)+R_{y_{I}}\left(\alpha ;\tau \right)+R_{y_{N}}\left(\alpha ;\tau \right)\\ & =\lim_{T\to \infty }\frac{1}{T}\sum_{t=0}^{T-1}y_{S}\left(t\right)y_{S}^{*}\left(t+\tau \right)e^{-j2\pi \alpha t}\\ & =\langle \left[A_{r}^{2}e^{-j2\pi f_{r}\tau }+A_{h}^{2}e^{-j2\pi f_{h}\tau }\right]+\left[A_{h}A_{r}e^{-j2\pi f_{h}\tau }\right]e^{-j2\pi [\alpha -\left(f_{r}-f_{h}\right)]t}\\ & \;+\left[A_{r}A_{h}e^{-j2\pi f_{r}\tau }\right]e^{-j2\pi [\alpha +\left(f_{r}-f_{h}\right)]t}\rangle_{t}+R_{y_{I}}\left(\alpha ;\tau \right)+R_{y_{N}}\left(\alpha ;\tau \right) \end{array}$$

where $R_{y_{I}}\left(\alpha ;\tau \right)$ and $R_{y_{N}}\left(\alpha ;\tau \right)$ are respectively cyclic autocorrelation functions of the subject random movement and Gaussian noise. From (30), $f_{r}$ and $f_{h}$ are not still obtained by using the second-order cyclostationary method in Hilbert space.

For others harmonic signals (corresponding to frequencies $nf_{r}+mf_{h}$ in (20), the corresponding harmonic periodicity frequencies can be obtained by the aforementioned analysis in this Section.

## 3. Statistical Property Analysis of Higher Order Cyclostationary Detection

From the analysis in Section 2, the third-order cyclostationary property of the Doppler radar signal was proven in a time-series analysis. Next, we will analyse the statistical properties of the third-order cyclostationary detection method.

### 3.1. The Almost Sure Convergence of the Time Varying Cyclic-Moments and the Sample Cyclic-Moments

Let ${\left\{x_{m}\left(t\right)\right\}}_{m=0}^{k}$ be k+1 deterministic or random signals. An Assumption 1 [27] is given as follows.

Assumption 1: ∀m∈Z

$$\begin{array}{c} \sum_{\tau_{1}\cdots \tau_{m}=-\infty }^{\infty }\underset{t}{sup}\left|cum\left\{x_{n_{0}}\left(t\right),x_{n_{1}}\left(t+\tau_{1}\right),\cdots ,x_{n_{m}}\left(t+\tau_{m}\right)\right\}\right|<\infty \end{array}$$

where $x_{n}\left(t\right)\in \left\{x_{0}\left(t\right),x_{0}^{*}\left(t\right),\cdots ,x_{k}\left(t\right),x_{k}^{*}\left(t\right)\right\}$. If x(t) is a *k*-th order cyclostationary process, and which satisfies Assumption 1, with $x_{n}\left(t\right)\in \left\{x\left(t\right),x^{*}\left(t\right)\right\}$. Then for $\gamma >\frac{3}{4}$ , the third-order sample cyclic-moment of *x(t)* can be expressed as follow in [27]

(31) $${\bar{M}}_{3x}^{\left(T\right)}\left(\alpha ;\tau \right)\overset{\Delta }{=}\frac{1}{T}\sum_{t=0}^{T-1}x\left(t\right)x\left(t+\tau_{1}\right)x\left(t+\tau_{2}\right)e^{-j\alpha t}$$

satisfies

$$E|{\bar{M}}_{3x}^{\left(T\right)}\left(\alpha ;\tau \right)-E{\bar{M}}_{3x}^{\left(T\right)}{\left(\alpha ;\tau \right)|}^{2}\leq \frac{C}{T}$$

(32) $$E|{\bar{M}}_{3x}^{\left(T\right)}\left(\alpha ;\tau \right)-E{\bar{M}}_{3x}^{\left(T\right)}\left(\alpha ;\tau \right)|\;\overset{a.s.}{=}\;O\left(\frac{1}{T^{1-\gamma }}\right)$$

where *C* is a constant, and a.s. represents almost sure convergence. The convergence rate is given in (32). According to the analysis in Section 2, the $M_{3x}\left(\alpha ;\tau \right)$ exist, therefore we have follow in [27]

(33) $$\lim_{T\to \infty }{\bar{M}}_{3x}^{\left(T\right)}\left(\alpha ;\tau \right)\;\overset{a.s.}{=}\;M_{3x}\left(\alpha ;\tau \right).$$

Equation (33) implies the almost sure convergence of the estimator of the third-order time varying cyclic moments and the third-order sample cyclic-moment. Therefore, the third-order cyclic moments $M_{3x}\left(\alpha ;\tau \right)$ can be estimated by the third-order sample cyclic-moment ${\bar{M}}_{3x}^{T}\left(\alpha ;\tau \right)$, then we have

(34) $$\begin{array}{cc} M_{3x}\left(\alpha ;\tau \right) & \approx {\bar{M}}_{3x}^{\left(T\right)}\left(\alpha ;\tau \right)\\ & =\frac{1}{T}\sum_{t=0}^{T-1}x\left(t\right)x\left(t+\tau_{1}\right)x^{(}t+\tau_{2})e^{-j\alpha t}. \end{array}$$

From (10), (11), (12) and (34), the estimated third-order cyclic cummulant ${\bar{C}}_{3x}^{\left(T\right)}\left(\alpha ;\tau \right)$ can be obtained as

(36) $${\bar{C}}_{3x}^{\left(T\right)}\left(\alpha ;\tau \right)={\bar{M}}_{3x}^{\left(T\right)}\left(\alpha ;\tau \right).$$

However, in (35), *T* is the finite number of sample elements. Therefore, we have

(36) $${\bar{C}}_{3x}^{\left(T\right)}\left(\alpha ;\tau \right)=C_{3x}\left(\alpha ;\tau \right)+\varepsilon_{3x}^{\left(T\right)}\left(\alpha ;\tau \right)$$

where $\varepsilon_{3x}^{\left(T\right)}\left(\alpha ;\tau \right)$ is the estimation error. Due to this error, the estimator ${\bar{C}}_{3x}^{\left(T\right)}\left(\alpha ;\tau \right)$ is seldom exactly zero in practice when α is not a cycle frequency. Therefore, to determine whether a given value of ${\bar{C}}_{3x}^{\left(T\right)}\left(\alpha ;\tau \right)$ is zero, the hypotheses statistical testing approach in [28] is adopted in this work.

### 3.2. The Relation Analysis Between the Finite-Time Average and the Ensemble Average

For a time-series $\vec{x}$, according to [25], the third-order faction-of-time (FOT) probability distribution function is defined by

(37) $$F_{\vec{x}}\left(\vec{y}\right)\;\overset{\Delta }{=}\;{\bar{E}}^{\left(\alpha \right)}\left\{\prod_{i=1}^{3}U\left[y_{i}-x\left(t+t_{i}\right)\right]\right\}$$

where

$$\vec{y}\;\overset{\Delta }{=}\;{\left[y_{1},y_{2},y_{3}\right]}^{T},\vec{x}\;\overset{\Delta }{=}\;{\left[x\left(t+\tau_{1}\right),\cdots ,x\left(t+\tau_{3}\right)\right]}^{T},$$

and ${\bar{E}}^{\left(\alpha \right)}\left\{\cdot \right\}$ is the multiple sine-wave extraction operation. U[·] is simply the event-indicator function. Therefore, by the *n*-fold derivative of the distribution function, the FOT probability density function for x(t) is written as

(38) $$f_{\vec{x}}\left(\vec{y}\right)\;\overset{\Delta }{=}\;\frac{\partial^{3}}{\partial y_{1}\partial y_{2}\partial y_{3}}F_{\vec{x}}\left(\vec{y}\right).$$

Assumed $g[\vec{x}]$ is a function of the vector of time-samples, and redefine ${\bar{E}}^{\left(\alpha \right)}\left\{\cdot \right\}$ to be the expected value with respect to the FOT $f_{\vec{x}}\left(\vec{y}\right)$. Accordingly, we can obtain as follow in [25]

(39) $$\begin{array}{cc} {\bar{E}}^{\left(\alpha \right)}\left\{g\left[\vec{x}\right]\right\} & \overset{\Delta }{=}\;\int_{-\infty }^{\infty }\int_{-\infty }^{\infty }\int_{-\infty }^{\infty }g\left[\vec{y}\right]f_{\vec{x}}\left(\vec{y}\right)d\vec{y}\\ & \overset{\Delta }{=}\;\sum_{\alpha }\langle g\left[\vec{x}\right]e^{-j2\pi \alpha t}\rangle e^{j2\pi \alpha t} \end{array}$$

where 〈·〉 is the time-averaging operation. The ensemble average can be defined through the finite-time average of a single time series in higher order cyclostationary analysis in (39).

Based on the above statistical properties of the third-order cyclostationary detection method, the statistical significance of estimated frequencies of the heartbeat and respiration can be analysed when the Probability of False Alarms (PFA) is a constant value.

## 4. Experiment and Analysis

In order to demonstrate the effectiveness and robustness of the proposed method (third-order cyclic cumulant (TOCC)) to detect the heartbeat and respiration rates, four experimental examples are given.

### 4.1. The Detection of the Heartbeat and Respiration Rate under Different SNR for Simulation Signals

In the first example, the respiration frequency and the heartbeat frequency are detected with signal to interference ratio (SIR) and different signal to noise ratio (SNR) for the simulation model in (5). We define SNR as the ratio of the signal power to the sum of the power of receiver noise and phase noise Δϕ(t) , and the SIR as the ratio of the signal power to the power of the motion interference $x_{I}\left(t\right)$, which is assumed a uniform random process. Here, carrier frequency of Doppler radar is 10.587 GHz (λ≈2.8 cm), and the $a_{h},a_{r},f_{h}$ and $f_{r}$ values in this example are assumed as 0.06 cm, 0.2 cm, 1.3 Hz and 0.5 Hz, respectively. The receiver noise and phase noise are assumed Gaussian noise which is obtained by filtering a white Gaussian noise through a $y\left(z\right)=sqrt\left(1-a^{2}\right)/\left(1-a\times z^{-1}\right)$ first order filter, where *a* is a coefficient. SNR and SIR are −22 dB and −2 dB, respectively.

**Figure 4:**
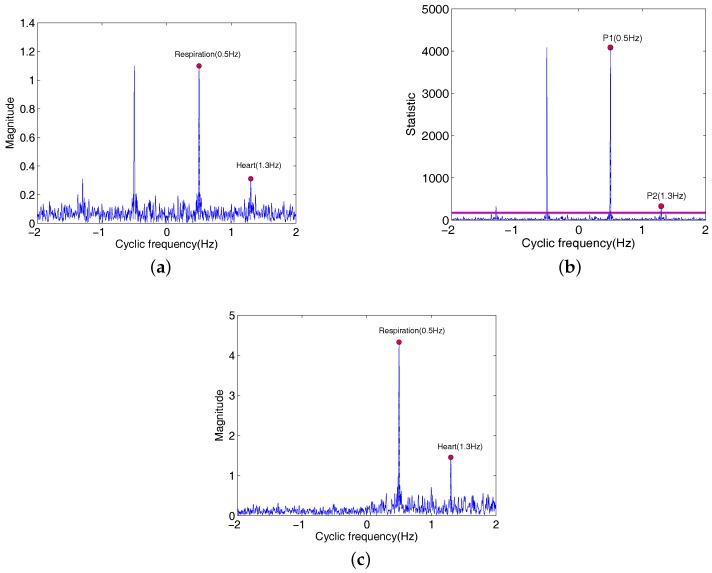
TOCC of the received signal. (**a**) TOCC of the received signal; (**b**) Test statistic (purple line denotes the threshold); (**c**) TOCC of the received signal in Hilbert space.

The TOCC of the signal B(t) in (5) are shown in Figure 4. The respiration and heart frequencies extracted are equal to 0.5 Hz and 1.3 Hz, respectively. This suggested that this received signal has a third-order cyclostationary nature, which is insensitive to all of the noncyclic components and Gaussian noise. From Figure 4b, only two peaks P1 and P2 corresponding to frequencies 0.5 Hz and 1.3 Hz are statistically significant when the Probability of False Alarms (PFA) in [28] is 0.001.

In Figure 4c, it shows TOCC of the received signal in Hilbert space. It is obviously to single sideband for the first harmonic after performing Hilbert transform for the received radar signal. Comparing with Figure 4a, the estimation precisions of respiration and heart frequencies have not improved apparently in Figure 4c. In fact, in almost all cases, the original third harmonic and the introduced signal $3f_{r}$ are usually different in phase due to noise, and these harmonics may suppress each other. In addition, the energy of the third harmonic is very little and the improved harmonic is usually hidden in the strong background noise.

**Figure 5:**
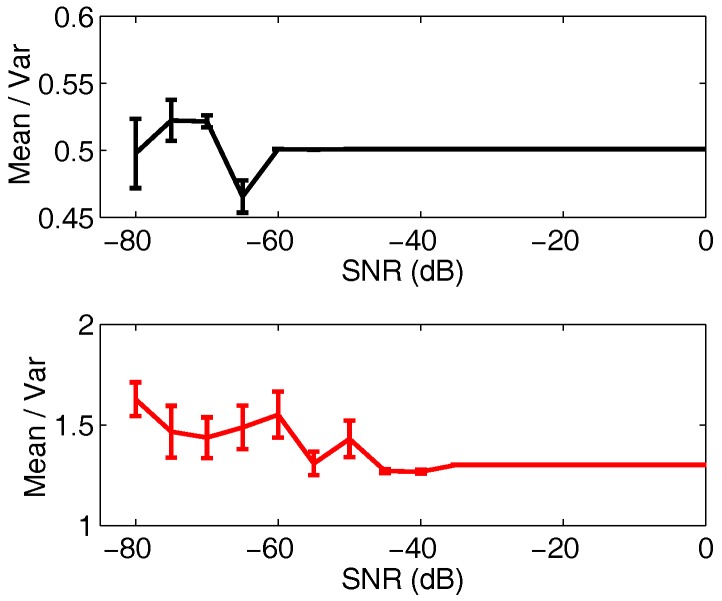
Estimated means and variances of the heart and respiration for 20 realizations of TOCC in each SNR levels (SIR = 0 dB).

As the subject movement $x_{I}\left(t\right)$ cancellation have been discussed in detail in [21], here we mainly concern on the robustness of the third-order cyclostationary method to vital-sign detection in the heart and respiration at various Gaussian SNR levels. In Figure 5, it shows the estimated means and variances of the heart and respiration for 20 realizations of the proposed approach in each SNR levels when the value of SIR is 0 dB. Simulation results indicate that the third-order cyclostationary approach is insensitive to Gaussian noise and can accurately detect the heart and respiration information even with low SNR. It is also noted that the estimation error of the heart beat is much bigger than the respiration due to the small chest movement.

### 4.2. The Detection of the Heartbeat and Respiration Rate Using the Doppler Radar Signal for a Single Subject

**Figure 6:**
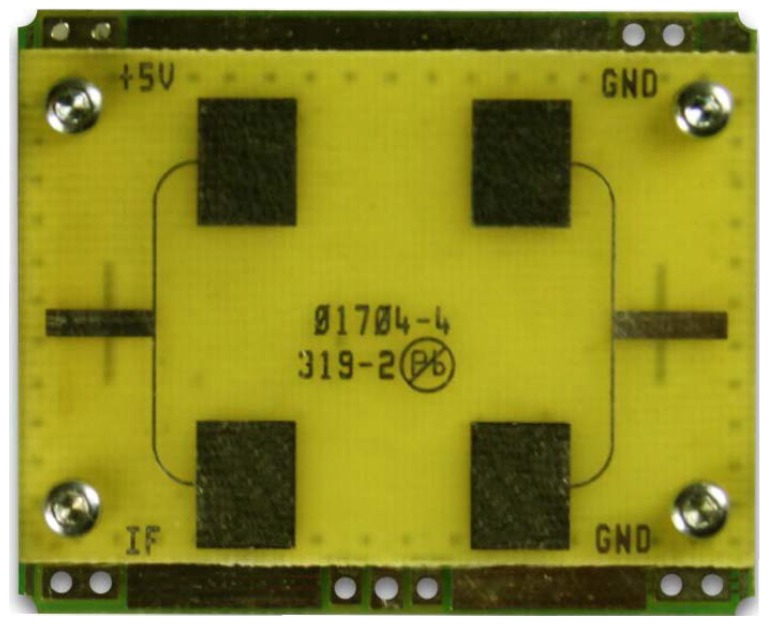
Doppler radar Motion Detector Unit (MDU1100T).

**Figure 7:**
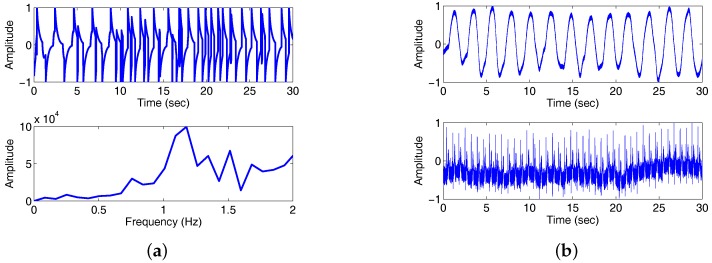
Doppler radar signal and reference signals ECG and RW. (**a**) Received radar signal (**top**) and power spectrum density (PSD) of the radar signal (**bottom**); (**b**) RW (**top**) and ECG (**bottom**).

In the second example, an Doppler Radar Motion Detector Unit (Figure 6) is used to get the vital sign data of a human subject. The detail parameters of the Detector Unit (Model number: MDU1100T, Manufacturers: Microwave Solutions Ltd.) are shown in Table 1. The radar to the human chest distance is 30 cm, and the data sampling rate is 44.1 KHz. The received radar signal plus the slight random subject movement and Fourier spectrum of the received signal in 30 seconds are shown in Figure 7a. The electrocardiogram (ECG) and respiratory waveform (RW) obtained by g.USBamp and g.TRIGbox are used as reference system to validate the measurement errors is shown in Figure 7b. We can obtain the respiration frequency 0.43 Hz and the heart beat frequency 1.4 Hz with the ratio of peak numbers to time (30 s). According to the aforementioned theory analysis, the received radar signal includes the frequency information of the respiration and heart beat. We apply the proposed third-order cyclostationary method to detect heart and respiration frequencies from the received signal.

**Figure 8:**
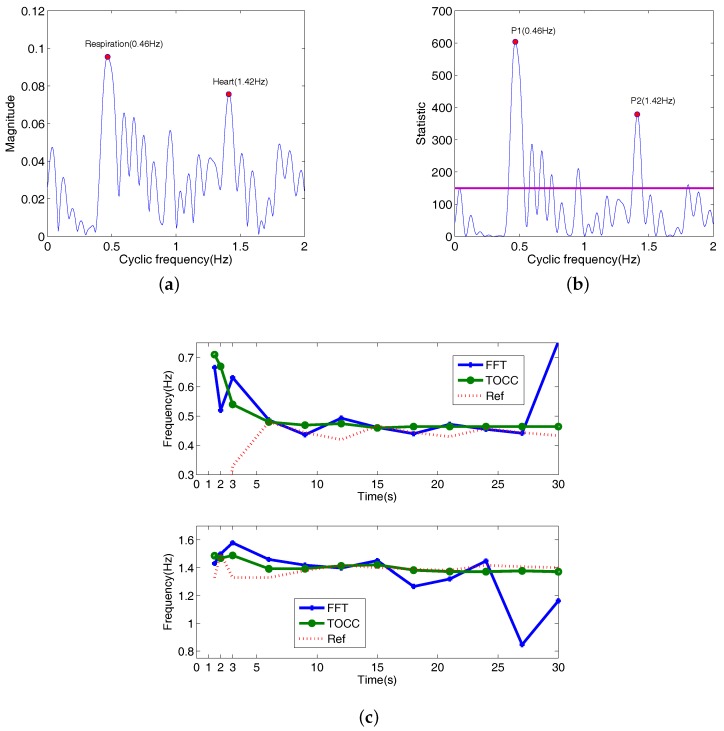
TOCC of the received radar signal. (**a**) TOCC 2-D graph; (**b**) Test statistic (purple line denotes the threshold, here PFA = 0.001); (**c**) The detected results of the heart and respiration rates with the record time increased in the 3 s steps (bottom: heart, top: respiration, Ref indicates the reference).

Figure 8 shows TOCC graph of the received radar signal. It is obvious seen that heart and respiration peaks corresponding to heart and respiration frequencies are shown in Figure 8a. According to (28), there are only frequencies of the respiration and heartbeat in the single sideband in Hilbert space. Respiration and heart frequencies obtained are equal to 0.46 Hz and 1.42 Hz in Figure 8a and are statistically significant at PFA = 0.001 in Figure 8b. Comparing with the reference frequencies in Figure 7b, the estimated results have only a little errors. However, from the Fourier spectrum of the received radar signal in the bottom of Figure 8a, it is difficult to obtain the two peaks corresponding to respiration and heartbeat frequencies.

In Figure 8c, it shows the detected results of the heart and respiration frequencies by using TOCC and FFT. An initial size of the 3 s time window which starts from zero is used to obtain the first measurement point by using the proposed TOCC method, and then 10 measurement points are obtained in 30 s (here FFT method: after performing FFT for the received radar signals, frequencies corresponding to maximum peaks in the range 0.1–0.8 Hz and 0.8–2 Hz are also considered as estimated frequencies of respiration and heartbeat signals, respectively). From Figure 8c, TOCC approach has better detection accuracy than conventional FFT method. Especially, for detecting heartbeat with FFT, when the frequency of the interference component belongs to the frequency range of the vital signs, estimated results gravely deviate from the reference in the 27th second and the 30th second. As the respiration has a bigger energy, the respiration frequency is detected accurately with both the Fourier and TOCC when the recording time reaches to the 6th second. The estimation performance of TOCC is improved with the recording time increased, (especially, when time is less than 9 s, the trend is more distinct) which it is consistent with the theory analysis. In addition, in order to test the estimation performance of the proposed approach for a short data sequence, the experiment results obtained by using the data corresponding to 1.5 s and 2 s are also shown in Figure 8c. Here, there is only a small estimation error for estimating the heart rate. However, the obtained respiration rates gravely deviate from the Ref (where Ref are consider as zero when the time is less than 3 s), because the time of duration of data is less than one period of the respiration signal, which can lead to the performance degradation for cyclostationary detection. From Figure 8c, based on the proposed method, the results estimated by dividing the time interval into much smaller intervals can be used to close to the real value of respiration rate and heart rate when respiration and heart rates keep changing.

### 4.3. The Detection of the Heartbeat Rate Using the Doppler Radar Signal for Different Subjects

**Figure 9:**
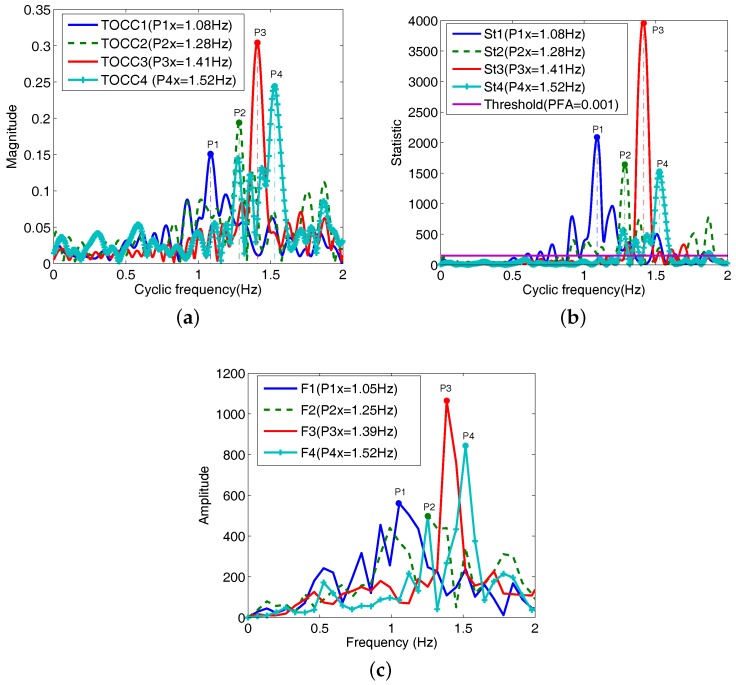
Estimated results of the heartbeat in the state of holding breath. (**a**) TOCC estimators of Subject1, Subject2, Subject3 and Subject3 plus motion; (**b**) Test statistic; (**c**) FFT spectrum corresponding to the Subject1, Subject2, Subject3 and Subject3 plus motion.

In the third example, the detection of heartbeat frequencies is studied when three persons are in the state of holding their breath. The chest subject faces the radar antenna at a distance of 30 cm. When the vital sign of Subject3 is recorded, the random subject movement is added by hand moving at an approximate constant speed in front of the radar system, and the subject movement magnitude is about 5 cm. The recording time of the valid data set is more 15 s in this case. The detected results of heartbeat and respiration rate are shown in Figure 9.

Here, heartbeat frequency references from ECG of Subject1, Subject2 and Subject3 are 1.13 Hz, 1.3 Hz and 1.4 Hz, respectively. From Figure 9a, there is no apparent peak in the frequency range (0.1–0.8 Hz) of the respiration due to persons holding their breath. Estimated heart frequencies of Subject1, Subject2, Subject3 and Subject3 plus the random movement are equal to 1.08 Hz, 1.28 Hz, 1.41 Hz and 1.52 Hz. Comparing with the ECG reference, estimation errors of heartbeat frequencies of three persons are very small. For the subject3 plus the random movement, the estimation error is less than 10%. Figure 9b shows test statistics St1, St2, St3 and St4 corresponding to Subject1, Subject2, Subject3 and Subject3 plus the random movement. It is clearly that heartbeat frequencies 1.08 Hz, 1.28 Hz, 1.41 Hz and 1.52 Hz are statistically significant at PFA = 0.001, and these frequencies are as expected in Figure 9a. It is also observed that the proposed TOCC method has detected the heart frequency with more clearly peaks compared to the Fourier transform shown in Figure 9c.

### 4.4. The Detection of the Respiration Rate Using the Doppler Radar Signal for Multiple Subjects

In the fourth example, the estimation of the respiration rate is analyzed with different respiration rates determined by a metronome. Four different tempos 40 bpm, 54 bpm, 56 bpm and 80 bpm are used, and four vital sign radar signals R1, R2, R3 and R4 corresponding to four tempos are recorded, and that the recording time of the valid data set continues 15 second for each tempo. Therefore, respiration reference frequencies of R1, R2, R3 and R4 are 0.33 Hz (40/(2 × 60) = 0.33), 0.45 Hz, 0.47 Hz and 0.67 Hz, respectively. The subject movement added in R2 is the same with the ones in the third example. In this case, the abdomen of the subject faces the antenna.

**Figure 10:**
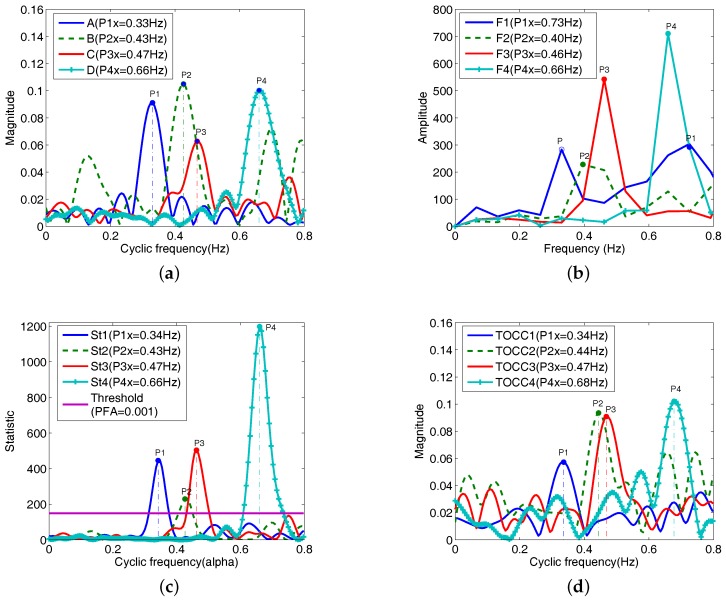

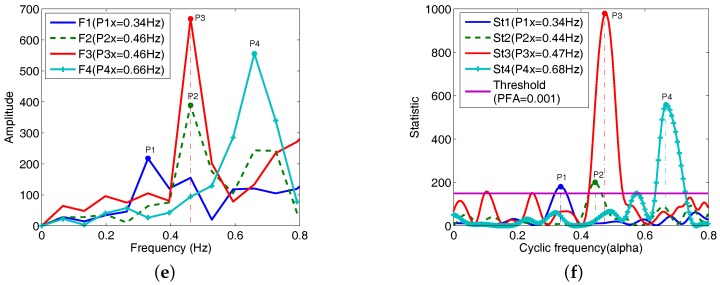
Estimated respiration frequencies and statistic of the received recorded respiration signal under the four different tempos. (**a**) TOCCs of R1, R2, R3, R4 (distance = 30 cm); (**b**) Fourier spectrum (distance = 30 cm); (**c**) Test statistic of (**a**); (**d**) TOCCs of R1, R2, R3, R4 (distance = 50 cm); (**e**) Fourier spectrums (distance = 50 cm); (**f**) Test statistic of (**d**).

TOCC estimators corresponding to R1, R2, R3 and R4 at a 30 cm distance between the antenna and the abdomen of the subject are plotted in Figure 10a. It is can be seen that estimated respiration frequencies of R1, R2, R3 and R4 are respectively 0.34 Hz, 0.43 Hz, 0.47 Hz and 0.66 Hz. A little estimation error is obtained by compared to the reference frequencies, and that comparing with Fourier transformation in Figure 10b, TOCC method has a more accuracy detection performance.

In Figure 10b, the respiration frequency should be obtained by the peak P, but the estimated result is the frequency 0.73 corresponding to P1. In Figure 10c, cyclic frequencies 0.34 Hz, 0.43 Hz, 0.47 Hz and 0.66 Hz corresponding to maximum peaks P1, P2, P3 and P4 are just statistically significant when the value of PFA is 0.001, which indicates the presence of cyclic frequencies which corresponding to maximum peaks in Figure 10a. From St1, the P1 in Figure 10b is not statistically significant. Therefore, the component corresponding to P1 in Figure 10b has no cyclostationary property at PFA = 0.001. Figure 10d shows the various plots analogous to the ones in Figure 10a. The distance between the antenna and the abdomen of the subject is 50 cm. As shown in Figure 10d, TOCC sidelobes of four radar signals are more than the ones in Figure 10a due to the increased distance between the antenna and the subject. However, due to bigger abdomen displacements caused by the respiration, estimated results of respiration frequencies are hardly affected by the increased distance. Although two respiration frequencies (0.45 Hz and 0.47 Hz) of R2 and R3 are closer each other, they are accuracy estimated by TOCC method. However, for using the FFT method, detected frequencies of R2 and R3 in Figure 10e are both 0.46 Hz. In Figure 10f, it shows the various plots analogous to the ones in Figure 10c. According to the Figure 10f, it is proved that estimated frequencies in Figure 10d are statistically significant when PFA = 0.001. From Figure 10, it can be seen that there is accurately estimation for the respiration frequency of R2 plus the subject movement. The effect of the subject movement can be reduced due to TOCC’s insensitive to the non-cyclic components.

## 5. Conclusions

In this paper, we have proposed a novel vital sign detection approach based on higher order cyclostationary. The respiration and heart frequencies can be accurately detected. Advantages of the new detection method, such as suppression of the noncylic components, high output SNR, cancellation of the subject movement, insensitivity of Gaussian signal, detection of weak signals, were analyzed in detail. These properties enhance the estimation accuracy of the heart and respiration rate when the detected subject lies in the complex environment with high noise and strong clutter. The theory and experiment results proved the second harmonic frequency and the interharmonic frequency can only be obtained by using the second-order cyclostationary method when the output signal of vital sign radar system is just proportional to the periodic subject displacement summed. By using the conventional FFT, the estimated result of the heart and respiration rate may be inaccurate due to the influences of the noncyclic component, clutter and noise. The third-order cyclostationary approach proposed has better detection accuracy under complex circumstance with strong clutter, noncyclic signal interference and Gaussian signals, which makes it a promising method to be used with Doppler radar to analyze the vital signs with the periodic motion information.

## Figures and Tables

**Table 1:** Main parameters for MDU1100T.

Components	Frequency	Power Output	Operating Voltage	Sensitivity	Gain	Noise
Specifications	10.587 GHz	10 dBm	+5 V ± 0.25 V	−86 dBm	8 dBi	<10 μV
